# Perspectives and Possibilities for New Antimicrobial Agents in the Treatment and Control of *Mastitis* Induced by Algae of the Genus *Prototheca* spp.: A Review

**DOI:** 10.3390/ijms25158219

**Published:** 2024-07-27

**Authors:** Weronika Jabłońska, Marcin Gołębiewski, Magdalena Kot, Henadzi Mardan, Bartosz Pawliński, Aleksandra Kalińska

**Affiliations:** 1 Department of Animal Breeding, Institute of Animal Sciences, Warsaw University of Life Sciences, 02-787 Warsaw, Poland; 2 Department of Large Animal Diseases and Clinic, Institute of Veterinary Medicine, Warsaw University of Life Sciences, 02-787 Warsaw, Poland

**Keywords:** algae, udder inflammation, nanobiotechnology, *Prototheca* spp., essential oil

## Abstract

Innovative approaches in nanotechnology provide a potentially promising alternative to untreatable cases of *mastitis* caused by genus *Prototheca* spp. algae infections. Drying of the teats of the affected animals or culling are typically the outcomes of *mastitis* in dairy cattle caused by these pathogens. A major issue in both veterinary medicine and animal breeding is the *Prototheca* species’ widespread resistance to the current methods of managing infections and the available drugs, including antibiotics. Commercial antifungal preparations are also ineffective. Nanotechnology, an emerging discipline, has the potential to create an effective alternative treatment for protothecal *mastitis*. The aim of the paper is to combine the literature data on the use of nanotechnology in the control of *mastitis*, taking into account data on combating *mastitis* caused by *Prototheca* spp. infections. The databases employed were PubMed, Google Scholar, and Scopus, focusing on literature from the last 20 years to ensure relevance and currency. Studies conducted in vitro have demonstrated that nanomaterials have significant biocidal activity against *mastitis* infections of different etiologies. Analyzed research papers show that (NPs), such as AgNPs, CuNPs, AuNPs, etc., may not negatively impact various cell lines and may be effective agents in reducing the pathogens’ viability. However, it is also critical to assess the risks involved in using nanomaterials.

## 1. Methodology of the Review

This review focuses on the use of nanotechnology in the treatment of *mastitis*, with a particular focus on infections caused by algae from the genus *Prototheca*. This literature review includes scientific papers from a variety of sources to ensure broad coverage and relevancy.

We used multiple databases to gather studies, including PubMed, Google Scholar, and Scopus. The following search terms were used: “*Prototheca* spp. management”, “*Prototheca* spp. *mastitis*”, “*mastitis* alternative treatments”, “nanotechnology in *mastitis*,” “nanoparticles *mastitis*”, “NPs treatment”, “nanoparticles–biocidal activity”, “nanotechnology *mastitis Prototheca*”, “nanoparticles in dairy cattle”, and “nanoparticles toxicology”. The focus was on literature published within the previous 20 years; therefore, the most recent and relevant findings were included. Our selection of studies was based on their applicability to the treatment of *mastitis*, especially those resulting from infections caused by *Prototheca* spp., using nanotechnology and other alternative compounds. We examined a variety of research, concentrating on the efficacy, safety, and possible risks of using nanomaterials in veterinary industries.

## 2. Introduction

Infections due to *Prototheca* spp., which is a chlorophyll-free unicellular algae, occur in both humans and animals. This review will have a particular focus on issues connected with cattle and milk production. The habitat of *Prototheca* spp. is wet and marshy locations, for example, bedding areas in the barns that are wet. They can also be found in animal feces and drinking water, or around water tanks [1].

Udder inflammation caused by *Prototheca* spp. results in increased economic losses for dairy farms due to the necessary disposal of the mastitic milk and cow culling. The higher culling rates in herds effected by *Prototheca* infections are a consequence of not utilizing efficient methods to control this pathogen. From an economic point of view, keeping infected animals in the herd is unfavorable; the infection also negatively influences milk production in cows diagnosed with protothecosis of the udder [2].

Another issue is the fact that infected cows can act as a vector and spread the pathogen to the healthy animals. Therefore, these infections pose a threat to all cows in the herd, especially young animals, which should be the main source of income for the farmer. Solutions for these cases, other than ineffective antibiotic treatments, are limited, which is a key problem. However, it is worth implementing the routine separation of infected cows and establishing a milking order so that infected animals are milked last [3]. This is important to mention because these strategies may significantly decrease the possibility of disease transfer to healthy cows while also limiting the spread of infection across the herd. Furthermore, continuous monitoring of animal health and early detection of illness are crucial elements of good herd management. This reduces economic losses while simultaneously guaranteeing animal wellbeing, which is important to breeders.

The species of the genus *Prototheca* spp. that demonstrate pathogenicity are *Prototheca zopfii*, *Prototheca wickerhamii*, *Prototheca blaschkeae*, and *Prototheca cutis* [4]. According to studies conducted in European countries and Japan, in most cases of algae-induced *mastitis*, the cause of infection was *P. zopfii* genotype II. These algae most often cause a subclinical form of *mastitis* in dairy cattle [3]. It has also been proven that the algae that penetrate mammary gland tissue cause chronic granulomatous inflammation [5].

Typically, *Prototheca* spp. algae develop small colonies that rarely get much bigger than 30 µm [6]. A saprophytic lifestyle is what they often lead [7]. Considering how similar their morphology is, they were previously categorized as fungi [8]. *Prototheca* was one of the principal subjects of medical mycology following Krüger’s classification of them. *Prototheca*, which is currently a member of the *Trebouxiophyceae* family of green algae, originated from the genus *Chlorella* nearly 498.5 million years ago. *P. wickerhamii* is an illustration of how a single strain has evolved; it originated from the genus *Chlorella* approximately 104.9 million years ago. The *Prototheca* genome changed over time in various forms. The modifications involved the emergence of genes linked specifically to drug resistance, bacterial disease transmission, or nutrition uptake*. Prototheca*, exactly like fungi, has evolved genes important for signaling pathways and post-translational modifications [6].

In addition, *Prototheca* spp. infections lead to an impaired host immune response, which results in frequent future relapses [9]. Results obtained by Shahid et al. also indicated that *Prototheca* strains, i.e., *P. zopfii* genotype II, can induce severe apoptosis in bovine mammary epithelial cells (BMEC) [10].

Since algae of the genus *Prototheca* resemble yeast in their morphology, they are often confused with yeast at initial identification. This has been indicated by both old and more recent reports [11,12]. The algae’s white to gray color and smooth colonies are similar in appearance to yeast [13]. *Prototheca* spp. have been categorized as a fungi belonging to the genus *Prototheca* based on genetic features [11,12]. Because the cause of *mastitis* varies depending on whether a herd is affected by a fungus or *Prototheca* spp., accurately identifying the source of the *mastitis* is essential for ongoing management.

Several approaches are, therefore, used to determine the cause of *mastitis*. Even hard-to-culture and possibly inert *mastitis* pathogens can be swiftly and reliably identified using the quantitative PCR (qPCR) technique. Studies on milk quality benefit from this technique’s ability to analyze multiple samples and, thus, shorten analytical times. It can be used to identify factors that cause inflammation and, therefore, treat cows selectively. Micro-organisms from bulk milk can also be identified using qPCR [14,15,16,17]. Because PCR can quickly and accurately identify infections over a broad scale, even at the subspecies level, it is useful in epidemiology and diagnostics. Additionally, it can track the source of infection, identify the pathways of transmission, and compute the infectivity of strains in epidemiological research on intramammary infection (IMI). Keeping an eye on drug-resistant strains helps with diagnosis [18].

The identification of algae was previously carried out using microscope images. The morphology of *Prototheca* spp. cells was studied by performing wet smears using Lugol’s solution. Smears were made using Lugol’s solution as well as various other staining methods, such as methylene blue, Congo red, fuchsin, and malachite green [19].

Currently, the identification of *Prototheca* spp. strains is based on several methods, including a comparison of 18S ribosomal DNA, amplification of the entire ITS 32 region, detection of the mitochondrial CYTB gene that encodes cytochrome b, and MALDI–TOF MS (matrix-assisted laser desorption ionization–time of flight mass spectrometry) [4,20,21].

An important aspect affecting the resistance of this species to known pathogen treatments is the presence of sporopollenin in the cell walls [22]. Sporopollenin has a low sensitivity to biological and chemical actions, as well as to acetolysis and is a robust heteropolymer dominated by ester and ether bonds [23].

The aim of this paper is to present the current state of knowledge and to attempt to evaluate the potential of nanobiotechnology advances in the prevention and control of *mastitis* in cattle caused by algae from the *Prototheca* genus.

## 3. Cost Analysis and Treatment Strategies for *Mastitis*

The costs associated with subclinical and clinical *mastitis* are divided into direct and indirect costs. Direct costs include expenses associated with drugs, labor, and rejected milk during treatment; while indirect costs include reduced milk production after treatment, culling, and mortality [24,25].

According to a work by Rodriguez et al., the estimated cost associated with subclinical *mastitis* that occurred during early lactation averaged USD170/cow. The cost range was from USD148 to USD187 per cow. For instance, an analyzed case of *mastitis* caused by *Streptococcus agalactiae* was estimated to be USD122/cow, with a range of USD101–USD148 for primiparous cows; and USD193/cow, with a range of USD163–USD221 for multiparous cows. They stated that clinical *mastitis* cases are typically the focus of cost analysis. Reports have indicated that the costs associated with a single case of clinical *mastitis* range from USD338 to USD594. Estimated losses are largely dependent on model assumptions, variable selection, direct and indirect costs, disease transmission, milk value, treatment costs, and herd parity structure [25]. Litwińczuk et al. estimated the average cost of *mastitis* incidence to be even up to €1000/cow/year [26]. There are similar figures in other European countries, in which total *mastitis* costs have been estimated to be in the range of €261 to €483 per cow per year [27,28]. In contrast, estimated average *mastitis* losses in the US are at a minimum of $131/cow/year [29].

Enhancing the efficacy of curative and preventive strategies for *mastitis* control is critically important given rising consumer concerns about animal health and welfare and the mounting financial strain on production expenses. To achieve this, it is necessary to carry out a detailed evaluation of the existing circumstances of a given farm, and have knowledge of its general operating environment [28]. In a study by de Campos et al., the prices of various *mastitis* treatments were calculated. These calculations showed that the range of expenses was from USD79.01 (treatment-free) to USD240.03 (combination therapy regimens) for antibiotics and supportive formulations. The study demonstrated that regulating the period of therapy can lower treatment costs, but also that treatment costs rise with increased milk supply. The study’s findings emphasized that, in order to lower the expenses connected with treating *mastitis*, it was necessary to base therapeutic decisions on the cause and modify the therapy duration accordingly [30].

The literature additionally reveals that there are economic benefits to using additional forms of *mastitis* treatment. The use of non-antibiotic formulations results in increased milk output following therapy. Non-antibiotic preparations may also be a more cost-effective form of *mastitis* treatment since they eliminate the need to discard milk containing the drug (antibiotic) residue [28,31].

## 4. Treatment Methods for *Mastitis* Induced by Algae

The most common antibiotic therapy used against *mastitis* in cows is ineffective against infections caused by *Prototheca* spp. Surprisingly, studies show that the use of antibiotic therapy against algae, as well as intramammary treatment, results in a higher risk of *mastitis*. In addition, *Prototheca* spp. demonstrates a resistance to high temperatures, which means that the pathogen can only be removed from milk after ultra-pasteurization [32].

Research on the antibiotic resistance of *Prototheca* spp. strains was conducted by Morandi et al. by using the disk-diffusion method. The effects of nisin, lysozyme, and potassium-sorbate solution were also investigated. For nisin, which is a bacteriocin, the above study showed that 73.8% of the *P. zopfii* genotype II strains isolated during the study were susceptible to this sub-treatment, while the remaining strains were resistant to nisin. This result indicates that nisin, a bacteriocin product for the bacterium Lactococcus lactis, can potentially be used in the control of *P. zopfii*. Unfortunately, this in vitro study also showed that the tested strains showed resistance to lysozyme and potassium sorbate [33]. Morandi et al. proved that *Prototheca* spp. strains were resistant to most of the tested antibiotics. The strains were 100% resistant to ampicillin, aztreonam, cefepime, ceftazidime, chloramphenicol, ciprofloxacin, erythromycin, fosfomycin, mupirocin, nitrofurantoin, oxacillin, penicillin G, and piperacillin [33].

However, in order to develop therapeutic forms against strains of *Prototheca* spp. algae, current research is targeting other substances, such as essential oils and nanoparticles (NPs).

Essential oils are characterized by the synergistic effects of many of the compounds contained in their structures. However, their effectiveness against all species of *Prototheca* remains inconclusive. The activity of essential oils against *P. zopfii* and *P. blaschkeae* has been repeatedly studied. To date, it has been reported that oils from *Citrus paradisi* show effective biocidal properties against both strains [34]. Resistance studies involving *Prototheca* spp. have also been conducted against other antifungal substances; it has also shown resistance to agents such as fluconazole and caspofungin.

In comparison, *Prototheca* spp.’s resistance to voriconazole depends on the species. Voriconazole is a synthetic triazole antifungal drug that is used to treat systemic fungal infections in animals. It is used particularly when other antifungal agents, like fluconazole, are ineffective. Voriconazole inhibits fungal growth by disrupting the cell membrane. This action is achieved by inhibiting fungal 14-alpha-sterol-demethylase [35]. *P. zopfii*, which is often isolated from *mastitis*, shows resistance to voriconazole, while *P. wickerhamii* is sensitive to it [36]. Amphotericin B and posaconazole, as well as tea tree oil, proved to be the only effective preparations in the in vitro tests conducted by Tortorano et al. [36]. In the same experiment, growth inhibition was also observed after treating the strains with bergamot oil [36]. Similar results were obtained in another study by Jagielski et al., who also tested antifungal agents against *mastitis*-causing algae. In their study, *Prototheca* spp. growth was reduced by 97% after treatment with < 2 mg/L of amphotericin B. In the same study, echinocandins and flucytosine were confirmed as having an ineffective antimicrobial influence [37].

Collecting data on the activity of individual substances against *Prototheca* spp. isolates that induce *mastitis* is particularly important in order to conduct further studies on the effectiveness of available biocidal agents and also to develop new methods. A summary of the effectiveness and testing procedures of several antibiotic and antifungal substances against *Prototheca* spp. is presented in Table 1, taking into consideration previous issues.

## 5. Potential Application of Nanotechnology Solutions in Protothecal *Mastitis*

Nanotechnology is an innovative field of science, referred to as nanoscience, involving the use and appropriate transformation of materials at the nanometer scale, where the size of the matter being used is limited to 1 to 100 nm. The manipulation of the matter is performed at the molecular and atomic levels [38]. Materials that are at the nanoscale have a high specificity in relation to cells. The specificity of nanoparticles to cells stems from their ability to reach and penetrate specific tissues and target specific molecules within cells, making them particularly useful in the diagnosis and treatment of diseases such as cancer. Nanoparticles can inherit the antigenic characteristics of source cells and interact with specific cells while avoiding being cleared by the monocyte/macrophage system. In the context of *mastitis*, nanoparticles coated with neutrophil membranes can prolong their action in peripheral blood and enhance interactions with damaged tissue in the udder. Neutrophil-coated nanoparticles can better interact with endothelial cells in infected areas due to increased adhesion to molecules [39,40]. It is because of these characteristics that nanomaterials are used to develop new drug delivery systems. This is due to the fact that they are neutral carriers and are able to bind to active biomolecules and rapidly target agents through a number of chemical interactions [41,42]. Hence, nanoparticles (NPs) can be used to develop gene therapy agents or improve magnetic resonance methods as signaling pathways [43]. Nanotechnology’s broad spectrum of applications also includes activities involving the diagnosis of pathogenic microorganisms through the use of nanochips and nanobiosensors [44]. Silver NPs (AgNPs) have found application also designing polymeric nanobiocomposites that can be similar material to the human tissues [45] or in contact lenses and other several applications [46,47]. AgNPs continue to have antibacterial properties when embedded in a variety of other materials, including acrylic resins and titanium coatings. Silver nanowires can be used to cover various surfaces, giving them antibacterial and antiviral characteristics and, thus, protecting them against bacteria [48]. Furthermore, AgNPs have found application in bioengineering for the creation of dental prostheses, contact lenses, corneal implants, and breast cancer treatments [49].

NPs are also used in the broad field of microbiology. Polymer NPs, as well as carbon nanotubes and metal NPs, have high antimicrobial properties and can significantly reduce the viability of different pathogens, for example, bacteria and, very promisingly, also fungi and algae [50,51]. 

The biocidal effects of NPs against microorganisms include inhibiting enzyme activity on the microorganisms’ surface and the ability to penetrate cell walls. NPs have also destabilized cell membranes and interfered with DNA replication and RNA synthesis in bacterial and fungal cells [52].

NPs use several mechanisms to achieve biocidal effects against microorganisms, such as the previously mentioned ability to penetrate cell walls. It is specifically the small size of these nanomaterials that makes penetration of the cell walls and cell membranes of microorganisms possible. After breaching the cell membrane, the nanoparticles effectively change the environment inside the cell [53]. As stated earlier, NPs can cause the destabilization of cell membranes, which is achieved through a mechanism that allows nanoparticles to bind electrostatically to cell walls. The cell barrier is disrupted, and, thus, its transmission systems become damaged [54]. The intracellular environment also undergoes changes, such as changes in the amount of cytoplasm, a decrease in cell wall-building substances, and the loss of protective substances. Cell structures can deform and change their shapes [55], while an increase in glucose levels and the swelling of organelles in fungal cells has also been observed [56].

NPs can interfere with DNA replication and RNA synthesis in bacterial and fungal cells [52], and, through the action of NPs, chromatin damage can also occur [56]. It has been shown by Slavin et al. that nuclear condensation causes damage to DNA. Nuclear condensation is the process by which chromatin, a complex of DNA and proteins within the nucleus, becomes densely packed. This condensation, which affects normal DNA function and gene expression, is frequently related to cell failure and apoptosis. Once inside, these ions can cause significant damage to cellular components, including DNA. AgNPs have been shown to cause DNA condensation and fragmentation in the nuclei of *Candida albicans*, a type of fungal cell [56]. Studies confirm that silver nanoparticles can react with proteins in pathogens’ structures, releasing silver ions that penetrate the cells [57]. Production of reactive oxygen species (ROS) by NPs, including CuNPs, leads to protein oxidation and the inhibition of cell proliferation [58,59].

In addition, the use of NPs in combination with available antibiotics increases the effectiveness of infection treatment due to increased absorption and the biodistribution of the biocide [60,61].

Through using AgNPs as an example, it has been proven that by releasing silver ions (Ag+), NPs are capable of inhibiting the growth of microorganisms. This action is based exactly on the interaction between the metal ions and the negatively charged cell walls of microorganisms. The released metal ions inhibit the action of cellular enzymes [62].

Significantly, the antimicrobial activity of NPs is also correlated with the shape and size of the nanomaterial. Cube-shaped NPs have been shown to result in high antifungal activity, as have sphere-shaped particles. In addition, having the correct size of NPs (i.e., small size) could mean easier diffusion, which is closely related to the potentially higher surface-to-volume ratio [63]. Metal NPs’ high surface-to-volume ratio is the main attribute that defines their antimicrobial capabilities. It is because of this property that NPs interact effectively with the cell membranes of microorganisms [64]. Chemical reactions occur on the surface of the substance; therefore, larger surface areas result in more reactions, making them more effective.

The reactivity of the metals is also dependent on their size, as exemplified by AuNPs being highly reactive at the nanometer scale, but not showing any reactivity at the macro-metric scale [65].

Nanotechnology revolutionizes material synthesis and device manufacturing, with nanoparticle synthesis being a vital aspect of this [48,66]. Temperature, reagent concentration, reaction duration, and pH all influence the nucleation and generation of stabilized nanoparticles. The generation of nanoparticles is strongly influenced by reaction time, and, in certain situations, fast changes can be seen that only take a matter of minutes. Temperature is also an important factor in determining production and structure [67]. These synthesis-dependent variations extend to the antimicrobial properties of nanoparticles (NPs). For instance, chemical reduction or green synthesis approaches improve NPs’ surface reactivity and antibacterial capabilities [68]. Thermal decomposition, another synthesis technique, affects NPs’ crystalline structure and stabilization, crucial for antimicrobial efficacy [69]. The polyol method, which involves dissolving metal precursors at low temperatures, is an adaptive methodology for nanoparticle creation. With strong antibacterial properties, it generates a variety of nanoparticle kinds, such as magnetic and metal oxide nanoparticles [67].

Antimicrobial activity is exhibited by NPs from many types of metal, including the most commonly used types, such as AgNPs, AuNPs (gold NPs), and CuNPs (copper NPs); but also zinc oxide NPs (ZnO-NPs), titanium dioxide NPs (TiO2NPs), nitric oxide releasing NPs (NO NPs), magnesium oxide NPs (MgONPs), and iron NPs (FeNPs) [55,59,63].

The use of metal NPs as pathogen-fighting agents holds promise for both human and veterinary medicine, as microorganisms have not developed resistance to them [65]. This is a unique and important feature of NPs because pathogenic microorganisms’ resistance to commercial treatments is extremely dangerous and leads to global public health risks and results in non-optimal and ineffective treatment and the occurrence of epidemics [70].

However, it has recently been found that, with prolonged exposure, bacteria might develop resistance to AgNPs. Flagellin, a bacterial protein that causes the aggregation of NPs and lessens their antibacterial efficacy, is thought to be the source of this resistance. The process is based on phenotypic changes rather than genetic modifications. This form of resistance cannot be overcome by stabilizing AgNPs with surfactants or polymers; although, it can be mitigated by reducing flagellin synthesis [71]. However, it is important to emphasize that this research is relatively recent and focuses on specific bacteria. Amaro et al. maintain that detoxifying NP-generated reactive oxygen species (ROS), improving efflux, and decreasing NP absorption are examples of individual defense tactics. In order to prevent NP intrusion, bacteria may suppress porins, and, in order to release metal ions, they may upregulate efflux pumps. These modifications are seen in clinically relevant bacteria exposed to NPs [72].

To find out if resistance to NPs may be developed over time, *Staphylococcus aureus* strains were exposed to low quantities of AgNPs and AuNPs in a study conducted by Elbehiry et al. [73]. Ten strains were shown to be resistant to 20 nm AgNPs, whereas four strains demonstrated high resistance to 10 nm AgNPs. On the other hand, just two developed resistance to 10 nm AuNPs. Following ten consecutive passes in the absence of NPs, the acquired resistance remained stable across all adapted *S. aureus* strains. In the case described, *S. aureus* cells were exposed to silver nanoparticles (AgNPs) and gold nanoparticles (AuNPs) for a certain number of passes. Cells that were able to survive and proliferate in the presence of these nanoparticles were able to gradually adapt to their presence. As a result, the cells may have developed defense mechanisms or genetic modifications that made them less susceptible to these substances. Overall, it was discovered that both the 10 nm and 20 nm AuNPs produced less resistance in most *S. aureus* strains than AgNPs, notably the 20 nm AgNPs, after *S. aureus* was exposed to both NPs over an extended period of time [73].

The effectiveness of CuNPs against Gram-negative and Gram-positive bacteria, including *Escherichia coli*, *S. aureus*, and *Pseudomonas aeruginosa*, was demonstrated by Theivasanthi and Alagar [65]. The authors of the study used the standard microbiological zone of inhibition (ZOI) test. Studies were also conducted on the biocidal effects of AgNPs after applying them to *E. coli*. The zone of inhibition for E. *coli* was 15 mm after the application of CuNPs [65].

**Figure 1:**
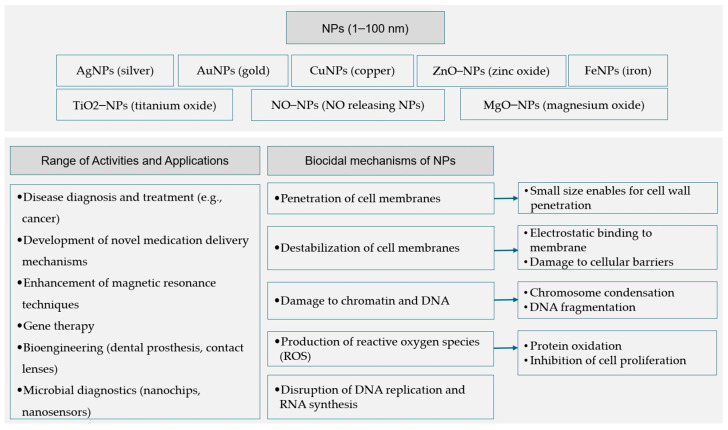
A general summary of the applications and mechanisms of action of nanoparticles utilized in biomedical research [35,36,37,38,39,40,41,45,46,47,48,49,50,51,52,53,54,55,56,57,58,59,60,61,62,63,64,65,66,67,68,69].

Orellano et al. established that chitosan nanoparticles (Ch-NPs), particularly those with a small diameter, prove to be beneficial in preventing *S. aureus* from forming biofilms and also in decreasing the number of bacteria that enter bovine epithelial cells. Because it inhibits bacterial virulence processes, this approach appears particularly promising in fighting infections [74]. Figure 1 summarizes the previously described uses and mechanisms of action for nanoparticles.

## 6. NPs as the New Antimicrobial Agents in Protothecosis Prevention and Treatment

A study by Fidelis et al. on *Prototheca bovis* isolates, showed that polyhexamethylene biguanide (PHMB) and chlorhexidine gluconate (CHG) had high antimicrobial efficacy, and that the formulations successfully achieved lower minimum inhibitory concentration (MIC) and minimum bactericidal concentration (MBC) values. For instance, MIC90 values for formulations against *P. bovis* were less than or equal to 2.0 µg/mL. Other agents, like sodium dichloroisocyanurate (≥700 µg/mL) and sodium hypochlorite (≥2800 µg/mL) exhibited higher MIC90 values than PHMB and CHG. Fidelis et al. suggested that PHMB and CHG may be effective options for treating *mastitis* [75].

The inhibition of microbial growth, according to literature data, correlates with increasing concentrations of NPs. This was demonstrated by decreased optical density and decreased sorption of the liquid bacterial medium [76]. In addition, it was proven that increasing the concentrations of AgNPs resulted in a decrease in the optical density of the medium. These results were also confirmed by another study using CFUs (colony-forming units) [77]. AuNPs also exhibited biocidal properties against *E. coli*, as demonstrated by an MBC/MIC ratio < 4 [77]. A study by Chwalibóg et al. proved that AuNPs effectively disrupt the cell walls of *Candida* spp. A biocidal effect is also possible after treating microorganisms with platinum NPs (PtNPs) [78]. Based on the literature, NPs also lead to the disintegration of algal structures, disrupting the proper functioning *Chlorella vulgaris* and *Dunaliella tertiolecta* [79]. A work by Hozyen et al. also demonstrated the antibacterial activities of ZnO-NPs against isolates of *S. aureus*, *E. coli*, and *Klebsiella pneumoniae*, which was proven by determining the average diameters in a ZOI test of a pattern of pathogens after the application of NPs. In addition, this work proved that the ZOI obtained during experimental activities for NPs was dependent on their concentration [80]. Elbehiry et al. provided evidence of the multi-complex efficacy of AgNPs, including their ability to efficiently battle multidrug-resistant bacteria. In this study, the AgNPs and AuNPs were found to be effective against *S. aureus* isolates using a serial dilution approach. The lowest concentrations required to eradicate the bacterium were 3.12–25 μg/mL for AgNPs and 6.25–50 μg/mL for AuNPs. All isolates were shown to be sensitive to the in vitro-tested NPs. Growth inhibition curves demonstrated that the particles were effective at concentrations between 0.39 and 200 μg/mL [73]. Most in vitro studies aimed at developing therapeutic methods against *Prototheca* spp. algae have used compounds such as commonly available antibacterial and antifungal agents, disinfectants, herbicides, and NPs [81,82].

However, there is not much research on the effectiveness of NPs against algae [83]. Studies have shown that *Prototheca* spp. have the ability to form biofilms, allowing clusters of cells from this pathogen to easily adhere to the surface of their host. Based on this finding, it can be hypothesized that metal nanoparticles may offer an effective approach to combatting *Prototheca* spp. strains by inhibiting their ability to form such biofilms [33,84,85]. Indeed, a study on the ability of AgNPs and CuNPs to inhibit biofilm formation by *mastitis*-inducing pathogens was conducted by Lange et al. [85]. The microorganisms used in the study were *Streptococcus agalactiae*, *Streptococcus dysgalactiae*, *Enterococcus faecalis*, *S. aureus*, *Salmonella enteritidis*, *E. coli*, *Enterobacter cloacae*, and *Candida albicans* [85]. The positive results obtained by the researchers may be the basis for similar studies on *Prototheca* spp. strains that form biofilms [86]. However, it is important to highlight that biofilms in real-world conditions are typically more complex than those observed in laboratory settings. These naturally occurring biofilms often comprise multiple types of pathogens, unlike controlled laboratory biofilms [87]. Radzikowski et al., carried out an investigation that examined the antibacterial properties of nanoparticles (NPs) on *mastitis* pathogens, such as *S. aureus*, *E. coli*, *Streptococcus* spp., and *Candida* spp. Iron nanoparticles with a carbon layer (NP-FeCs), AuNPs, CuNPs, AgNPs, PtNPs, and their complexes were evaluated. The authors also stated that the viability of bovine mammary epithelial cells (BME-UV1) was not significantly affected by NP treatments [88].

However, there are not many in vitro investigations on *Prototheca* eradication. And what is more important, and more surprising, there are, essentially, no studies conducted in vivo.

Jagielski et al. showed that seven out of the eight *Prototheca* genotypes tested responded to treatment using silver NPs, and that the MIC range was between 1 and 4 mg/L. They also demonstrated that AgNPs applied to *Prototheca* spp. did not have a drastic effect on algal cells, but that the ultrastructure of the algae was altered; and also, that the mass of the cytoplasm decreased and the cell membranes shrank [89]. Based on the considerations outlined above, a synthesis of the efficacy and testing methods of nanoparticles and other alternative substances across various pathogens has been compiled (Table 2).

## 7. Nanomaterials—Benefits and Risks

Due to the innovative solutions and wide range of possible applications of NPs and the promising prospects for their use in fields such as medicine, veterinary medicine, diagnostics, engineering, and biotechnology, it is crucial to closely evaluate their effects and mechanisms of action and thoroughly monitor their parameters. The advantages of NPs and potential risks connected with their practical applications should, therefore, be simultaneously determined.

To be able to assess their influence on living organisms, one topic that is popular among nanotechnology researchers is the cytotoxicity of NPs against various cell lines. The toxic effects of NPs can vary depending on the cell line being studied [83]. It has already been shown that the cytotoxicity of AgNPs at low concentrations is not great [78]. The mechanism responsible for the cytotoxic effect on cells is their ability to induce oxidative stress and activate the genes responsible for cell apoptosis. In the case of metal NPs, smaller particles are characterized by higher cytotoxicity. CuNPs have been proven to increase intracellular ROS levels, especially in mammalian cells [90].

According to results from several studies conducted by Jagielski et al. and Kalinska et al., the MIC level of AgNPs that inhibit the survival of *Prototheca* spp. was determined to be lower than that which causes cytotoxicity in bovine epithelial cells [89,91]. Kalinska et al. proved that AgNPs and CuNPs showed no cytotoxic effect against bovine or human mammary gland cell lines at lower concentrations, i.e., 0.1, 0.5, 1, 2, and 2.5 mg/L. The same authors also investigated the cytotoxicity of NPs in bovine epithelial cells and proved that AgNPs and CuNPs caused an increase in LDH enzyme levels [91]. Studies on cytotoxicity were also carried out by Sriram, who conducted an analysis of oxidative stress in bovine retinal endothelial cells (BREC) after treatment with AgNPs. The NPs caused ROS levels to increase in the cellular structure [92]. As stated by Paknejadi et al., even low concentrations of AgNPs can result in a cytotoxic effect in human fibroblasts, which was documented by exposing fibroblast cells to NPs for 48 h [93]. Additionally, the research demonstrated that in malignant HeLa cells, AuNPs and specific nanowires do not display appreciable toxicity [94]. According to the same study, 10 nm-diameter AgNPs did not negatively influence cells, but at 5.0 μg/mL, the NCTC 929 cell line and HepG2 cancer cells showed decreased viability. At 1.25 μg/mL, higher sensitivity was seen in the case of HepG2 cells. At all applied AgNP concentrations, the HeLa cells exhibited no response. The proliferation AgNPs has raised concerns regarding their health impacts, which emphasize how important it is to note that cytotoxic effects vary depending on the type of cells being studied [94].

Tang et al. carried out a focused investigation to look into the distribution and deposition of AgNPs in rat organs. The study’s findings showed that AgNPs were present in lung alveolar cells, indicating that they may be able to diffuse from the blood into the alveoli. The study demonstrated that AgNPs permeate into the alveoli from the lung capillaries, but more investigation is required to determine the mechanism underlying this phenomenon. The authors concluded by highlighting the advantages of nanotechnology for the environment and human health, but they also voiced concerns about the possible toxicity of AgNPs due to their capacity to cross cell membranes [95].

In addition, it is no secret that NPs can negatively affect the environment. Due to these possible negative effects, there is an ongoing effort to develop protocols for risk assessment, and regulations for the proper management of nanowaste. Ensuring the appropriate disposal of nanowaste is of paramount importance in mitigating its environmental impact and safeguarding ecosystems [87].

It should be stated that the topic of metal NP cytotoxicity is vast and complex. Because so much research is being conducted on the cytotoxicity of these structures, we are not able to say conclusively whether NPs have the potential to pose a threat or whether they are the future of science in a broader context.

In summary, however, the important point is that the cytotoxicity of NPs is a multifactorial parameter. The effect depends not only on the previously mentioned cell line, but also on the structure of the nanomaterial, the purpose for which the NPs are used, and many other factors. This makes the complexity of the field of nanotechnology unlimited at this point [57,83,96].

## 8. Conclusions

The use of NPs to monitor and control *Prototheca* spp. infections in bovine *mastitis* is still in the early phases of research. The efficacy of treatment can be impacted by variables like the NPs’ type, size, concentration, and application technique. Even though nanotechnology has great potential, employing metal NPs requires caution. Even at low doses, in vitro experiments, particularly those using AgNPs, are promising. Nonetheless, there are no in vivo investigations available, thus it is still unclear if NPs can be an effective agent against *Prototheca* algae in herd conditions. Further research on NPs is required to validate their efficacy, safety, and durability. Clinical trials and long-term impact research are required to fully evaluate NPs’ potential.

## Figures and Tables

**Table 1:** Summary of antimicrobial and antifungal efficacy against *Prototheca* spp. across various studies [33,34,35,36,37].

Pathogen	Type of Study	Substance(s) Used	Therapy Efficacy	Results	Source
*Prototheca* spp.	In vitro, MIC	Nisin, Lysozyme, Potassium Sorbate	Assessed resistance and susceptibility to different substances.	73.8% of *P. zopfii* genotype II strains were susceptible to nisin, while the remaining strains were resistant.All tested strains were resistant to lysozyme and potassium sorbate.	[33]
	Disk-Diffusion Method, antibiotic resistance study.	Ampicillin, Aztreonam, Cefepime, Ceftazidime, Chloramphenicol, Ciprofloxacin, Erythromycin, Fosfomycin, Mupirocin, Nitrofurantoin, Oxacillin, Penicillin G, Piperacillin	Evaluated resistance to a range of antibiotics.	100% resistance to tested antibiotics: ampicillin, aztreonam, cefepime, ceftazidime, chloramphenicol, ciprofloxacin, erythromycin, fosfomycin, mupirocin, nitrofurantoin, oxacillin, penicillin G, and piperacillin.	
*P. zopfii*, *P. blaschkeae*	In vitro, MIC	Citrus paradisi Oil	Assessed biocidal properties of essential oils against Prototheca spp.	Citrus paradisi oil showed effective biocidal properties against both *P. zopfii* and *P. blaschkeae.*	[34]
*Prototheca* spp.	In vitro, MIC	Fluconazole, Caspofungin, Voriconazole	Evaluated resistance to various antifungal agents.	*Prototheca* spp. showed resistance to fluconazole and caspofungin. Resistance to voriconazole varies by species: *P. zopfii* is resistant, while *P. wickerhamii* is sensitive.	[35,36]
		Amphotericin B, Posaconazole, Tea Tree Oil, Bergamot Oil	Evaluated effectiveness of antifungal agents and essential oils.	Amphotericin B and posaconazole were effective. Tea tree oil and bergamot oil also showed growth inhibition.	[36]
		Amphotericin B, Echinocandins, Flucytosine	Assessed growth reduction and effectiveness.	Growth was reduced by 97% with <2 mg/L of amphotericin B. Echinocandins and flucytosine were ineffective.	[37]

**Table 2:** Summary of nanoparticle efficacy and other alternative substances across various pathogens [65,73,74,75,77,78,79,80,85,88,89].

Pathogen	Type of Test	Substance(s) Used	Therapy Efficacy	Results	Source
*E. coli*, *S. aureus*, *Pseudomonas aeruginosa*	Zone of Inhibition (ZOI) Test.	CuNPs AgNPs	Assessed effectiveness against Gram-negative and Gram-positive bacteria.	Effective against *E. coli*, *S. aureus*, and *P. aeruginosa*	[65]
				ZOI for *E. coli* with CuNPs: 15 mm	
*S. aureus*	Resistance Development Study over Consecutive Passes.	AgNPs, AuNPs.	Assessed resistance development over time.	10 strains resistant to 20 nm AgNPs.	[73]
				4 strains resistant to 10 nm AgNPs.	
				2 strains resistant to 10 nm AuNPs.	
				Acquired resistance stable after 10 passes without NPs.	
				Less resistance to AuNPs compared to AgNPs.	
	Serial Dilution, Growth Inhibition Curves		Assessed effectiveness against multidrug-resistant *S. aureus.*	Effective concentrations: AgNPs: 3.12–25 µg/mL, AuNPs: 6.25–50 µg/mL	
				Effective at concentrations between 0.39 and 200 µg/mL	
*P. bovis*	Minimum Inhibitory Concentration (MIC) and Minimum Bactericidal Concentration (MBC) Tests	Polyhexamethylene Biguanide (PHMB), Chlorhexidine Gluconate (CHG), Sodium Dichloroisocyanurate, Sodium Hypochlorite	Assessed antimicrobial efficacy against *P. bovis.*	MIC90 for PHMB and CHG: ≤2.0 µg/mL	[75]
				MIC90 for Sodium Dichloroisocyanurate: ≥700 µg/mL	
				MIC90 for Sodium Hypochlorite: ≥2800 µg/mL	
				PHMB and CHG suggested as effective options for treating mastitis	
*E. coli*	MBC/MIC Ratio	AgNPs, AuNPs	Assessed biocidal properties of AgNPs and AuNPs.	AgNPs decreased optical density with increased concentration	[77]
				AuNPs: MBC/MIC ratio < 4 against *E. coli*	
*Candida* spp.	Nanoparticle-Microorganism Interactions with Hydrocolloid Preparation	PtNPs	Assessed disruption of cell walls.	AuNPs effectively disrupt cell walls of *Candida* spp.	[78]
				PtNPs potential biocidal effect	
*C. vulgaris*, *D. tertiolecta*	Algal Structure Disintegration	AgNPs,	Assessed disruption of algal structures.	NPs lead to disintegration of algal structures, disrupting function	[79]
*S. aureus*, *E. coli*, *K. pneumoniae*	Zone of Inhibition (ZOI) Test	ZnO-NPs	Assessed antibacterial activities and ZOI dependency.	ZnO-NPs: Antibacterial activity proven by ZOI; ZOI dependent on NP concentration	[80]
*S. agalactiae*, *S. dysgalactiae*, *E. faecalis*, *S. aureus*, *S. enteritidis*, *E. coli*, *E. cloacae*, *C. albicans*	Crystal Violet Staining Assay for Biofilm Quantification in a 96-Well Plate.	AgNPs, CuNPs	Assessed ability to inhibit biofilm formation in mastitis pathogens.	Positive results for inhibition of biofilm formation; suggests potential for similar studies on *Prototheca* spp.	[85]
*S. aureus*, *E. coli*, *Streptococcus* spp., *Candida* spp.	PrestoBlue Test, LDH Release Assay	Iron Nanoparticles with Carbon Layer (NP-FeCs), AuNPs, CuNPs, AgNPs, PtNPs	Evaluated antibacterial properties and cell viability.	- Evaluated multiple NPs; Viability of bovine mammary epithelial cells (BME-UV1) not significantly affected by NP treatments	[88]
*Prototheca* spp.	Minimum Inhibitory Concentration (MIC) Test, Ultrastructural Analysis	AgNPs	Assessed efficacy in treatment and cellular effects.	- MIC range: 1–4 mg/L against seven out of eight *Prototheca* genotypes	[89]
				- Ultrastructural effects: Decreased cytoplasm mass, cell membrane shrinkage	
